# Supplementary material for: Probing electronic-structure pH-dependency of Au nanoparticles through X-ray Absorption Spectroscopy
Source: Sci Rep. 2024 Dec 3;14:30059. doi: 10.1038/s41598-024-81580-y (PMC11615364; doi:10.1038/s41598-024-81580-y)
Supplement: Supplementary file 1 — Supplementary Material 1 [file 41598_2024_81580_MOESM1_ESM.docx]

*Supplementary Information for:*

Probing electronic-structure pH-dependency of Au nanoparticles through X-ray Absorption Spectroscopy

*Gabriela Imbir, Anna Wach, Joanna Czapla-Masztafiak, Anna Wójcik, Jacinto Sá, Jakub Szlachetko^*^*

*^*^e-mail:* [*jakub.szlachetko@uj.edu.pl*](mailto:jakub.szlachetko@uj.edu.pl)

Supplementary Figures S1 to S3 presenting XPS spectra of Au NPs

**
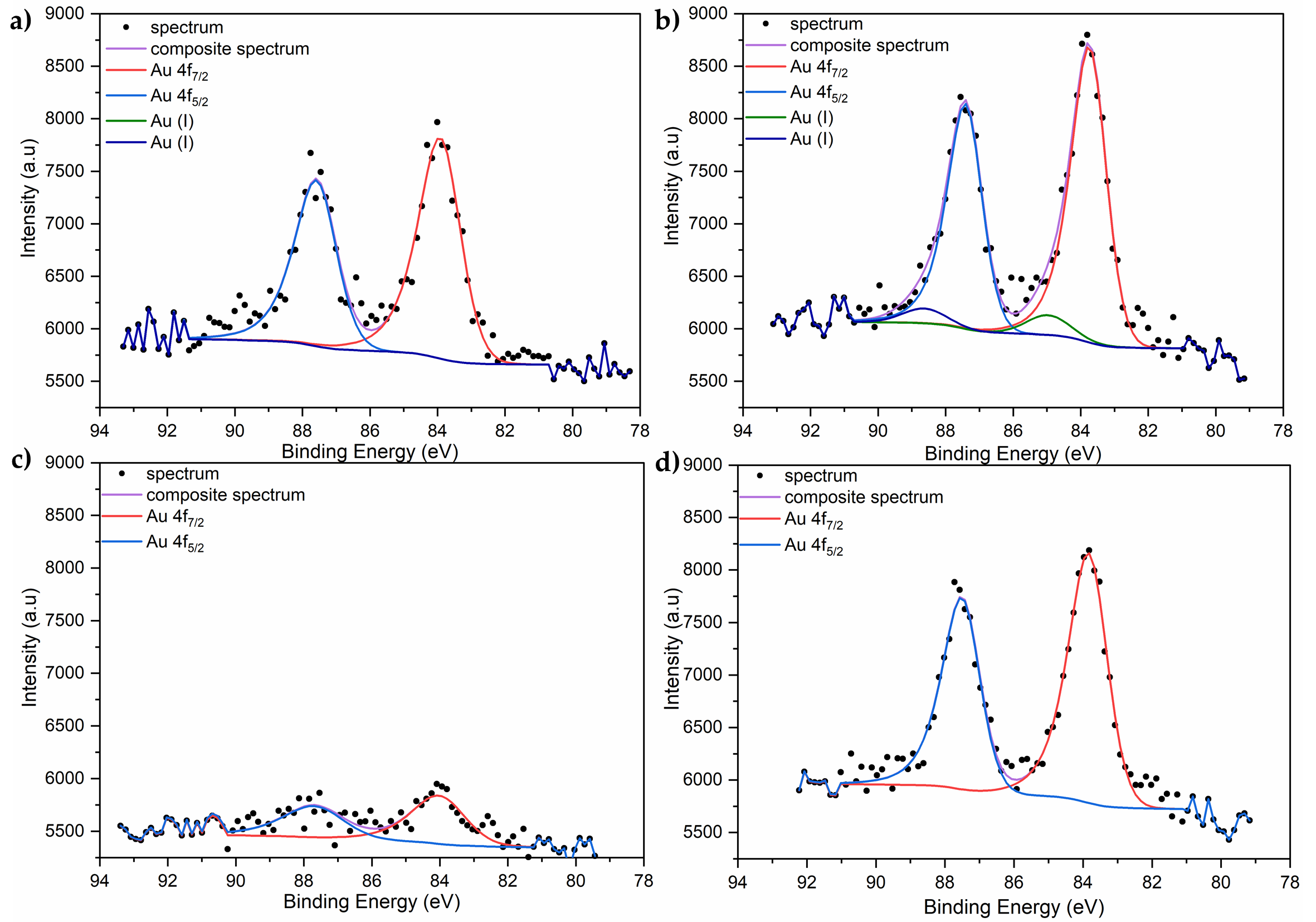
Figure S1.** XPS spectra of Au 4f binding energy for Au NPs: a) SC+TA, b) K_2_CO_3_, c) citric acid, d) 2 inj. seeding. Au (I) species confirms the carboxylate-gold coordination based on [].

**
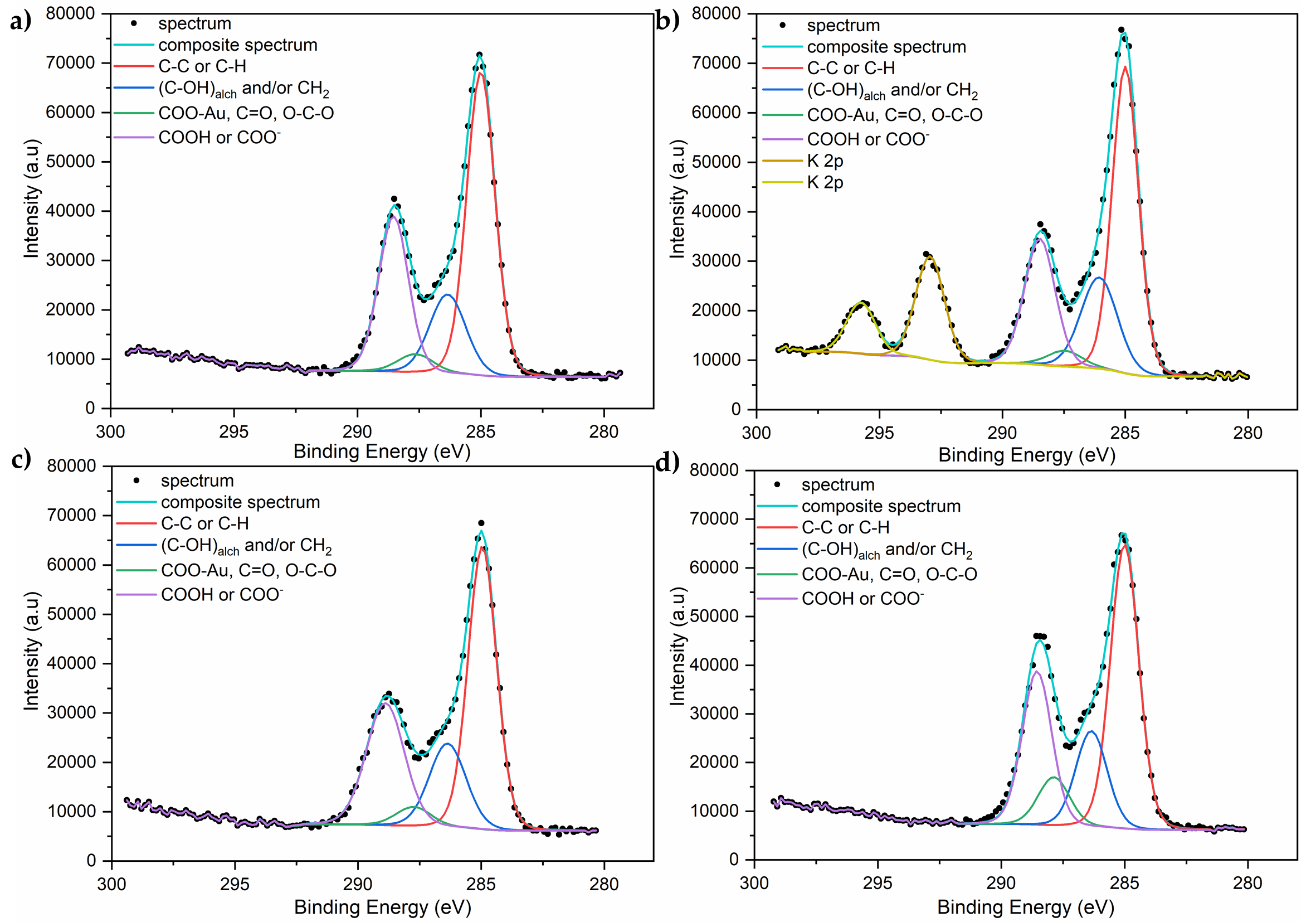
Figure S2.** XPS spectra of C 1s binding energy for Au NPs: a) SC+TA, b) K_2_CO_3_, c) citric acid, d) 2 inj. seeding. Spectra show coordination of COO^-^ groups on Au surface at 287.7 eV.

**
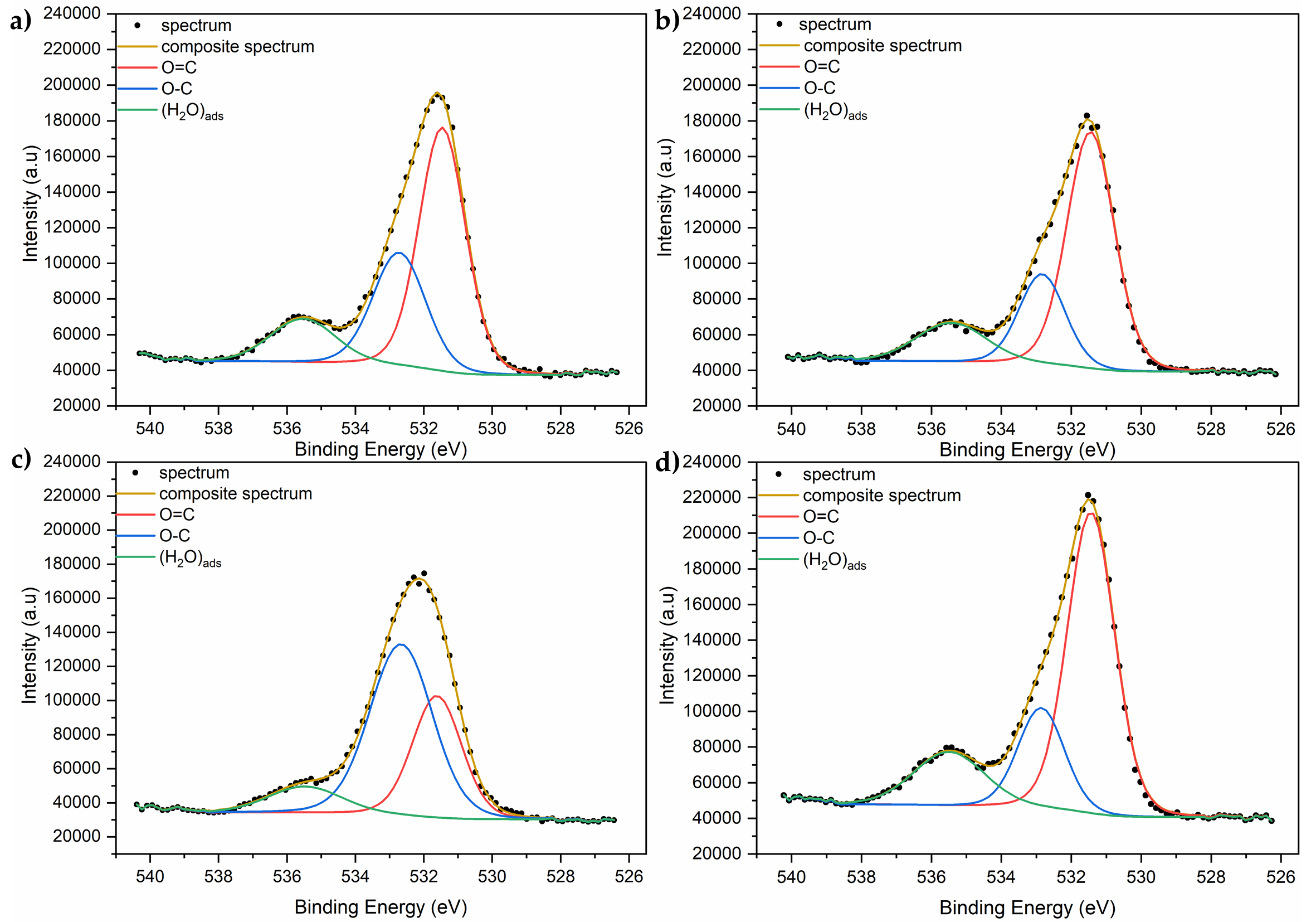
Figure S3.** XPS spectra of O 1s binding energy for Au NPs: a) SC+TA, b) K_2_CO_3_, c) citric acid, d) 2 inj. seeding. Spectra for Au NPs K_2_CO_3_ and 2 inj. seeding show high intensity of O=C groups derived from hydrogen bonds of COO^-^ .
