# Supplementary material for: Probing electronic-structure pH-dependency of Au nanoparticles through X-ray Absorption Spectroscopy
Source: Sci Rep. 2024 Dec 3;14:30059. doi: 10.1038/s41598-024-81580-y (PMC11615364; doi:10.1038/s41598-024-81580-y)
Supplement: Supplementary file 2 — Supplementary Material 2 [file 41598_2024_81580_MOESM2_ESM.docx]

**Probing electronic-structure pH-dependency of Au nanoparticles through X-ray Absorption Spectroscopy**

Gabriela Imbir^1,†^, Anna Wach^2,3,†^, Joanna Czapla-Masztafiak^1^, Anna Wójcik^4^, Jacinto Sá^3,5^, Jakub Szlachetko^2,*^

^1^Institute of Nuclear Physics, Polish Academy of Sciences, Radzikowskiego 152, 31-342 Krakow, Poland

^2^SOLARIS National Synchrotron Radiation Centre, Jagiellonian University, Czerwone Maki 98, 30 392 Krakow, Poland

^3^Institute of Physical Chemistry, Polish Academy of Sciences, 01-224 Warsaw, Poland

^4^Institute of Metallurgy and Materials Science, Polish Academy of Sciences, Reymonta 25, 30-059 Krakow, Poland

^5^ Physical Chemistry Division, Department of Chemistry, Ångström Laboratory, Uppsala University, 751 20 Uppsala, Sweden

^†^These authors contributed equally to this work

*e-mail: jakub.szlachetko@uj.edu.pl

**Abstract**

Research on gold nanoparticles (Au NPs) remains a field of intense activity due to their broad range of applications in diverse fields like catalysis, renewable energy, environmental science, and medicine. Herein, the morphological and electronic structures investigation of Au NPs prepared at different pH values is reported. The dependence of the localized surface plasmon resonance wavelength and electronic structure with size was determined by combining transmission electron microscopy, and various spectroscopic methods led by X-ray absorption spectroscopy. The X-ray absorption experiments evidenced that the citrate-stabilized Au NPs bulk electronic structure remains intact over a broad range of pHs, and changes were detected resulting from differences in NPs surface terminations.

**Keywords**: gold nanoparticles, physicochemical properties, pH dependency, x-ray absorption spectroscopy.

1. **Introduction**

Metal nanoparticles (NPs) represent a fascinating class of nanomaterials with a broad spectrum of applications in heterogeneous catalysis, sensors, photonics, batteries, and medicine [1]. Material properties at the nanoscale level are substantially different from the bulk counterparts due to their small size and high surface area. Moreover, the chemical reactivity of metal NPs is intimately linked to their elementary composition, atomic arrangement, shape, and size, which can be precisely controlled by synthesis conditions and appropriate functionalization. Nowadays, there is a lot of interest in nanoparticles within the small size range, typically less than 10 nm, as it becomes more feasible to precisely control their properties [2,3]. Smaller nanoparticles (Sub 10 nm) exhibit large surface areas with a significant number of surface atoms, which are chemically more active than the bulk ones. However, these nanoparticles tend to agglomerate when unsupported (uncoated), making it challenging to determine their structural and electronic properties [4].

Sub 10 nm Au nanoparticles (Au NPs) are recognized as exceptional nanostructures due to good physicochemical attributes, including surface reactivity, stability, and optical properties linked to localized surface plasmon resonance (LSPR). Obtaining well-controlled small-sized Au nanoparticles requires slow nucleation, where nuclei consist of a few Au atoms. These particles are commonly synthesized through homogeneous or heterogeneous nucleation by the reduction of gold salts (e.g., chloroauric acid, HAuCl_4_) with a reducing agent such as sodium borohydride, sodium citrate, and ascorbic acid [2,5]. Controlling the small-size range of Au NPs enhances their unique optical properties. According to Garcia et al., the surface plasmon effect for small-size range NPs (<50 nm) can be classified as intrinsic and extrinsic [6]. Generally, the intrinsic effect is related to the damping of electron oscillations, while the extrinsic effect corresponds to oscillating electrons scattering with the NPs surface. It was found that an increase in nanoparticle size causes a decrease in the electron fraction near the surface shell. Thus, damping is reduced [6], and consequently, a lower hot carrier formation [7].

Jeon et al. showed that the LSPR maximum exhibits a strong dependence on the shape of the Au NPs [8]. Others reported that the surface environment of Au NPs also affects the LSPR position since the capping agent interacts with the population of *d*-electrons on the surface [4,9]. More specifically, Au NPs capped with weak interacting agents can gain *5d* electrons and lose electrons when capped with strongly interacting molecules (e.g., thiols) [10]. It should be mentioned that when the Au NPs are brought into close proximity within an NP-size distance, the coupling effect from neighboring Au NPs is also very prominent, inducing changes in the plasmonic properties. Zhang et al. showed that reducing the size of nanoparticles affects the surface energy and the metal-absorbate or metal-substrate interaction, which, in consequence, has a notable impact on the electronic structure, especially of transition metals like gold [10,11]. Besides, it was found that increasing the concentration of the capping agent in Au nanorods leads to LSPR band redshift [12]. Therefore, many studies have shown that the electronic structure can be carefully controlled by the surface environment of metal NPs. The modulation of these characteristics is predominantly noticeable in the sub-10 nm range of nanoparticles. In this regime, *d*-*d* interactions in Au NPs may become more pronounced, leading to an increase in *d* charge at Au sites compared to the bulk [10].

The choice of the capping agent is a key factor not only for stabilizing nanoparticles but also for tailoring their properties to suit specific applications. The capping agent has a significant influence on the shape of nanoparticles, while its length, size, and chemical nature contribute to the stability of Au NPs. It was reported that different types of capping ligands, which particularly favor specific nanoparticle facets in NPs outgrowth, play a part in the LSPR shifting. If the ligands are composed of small molecules, the LSPR peak is blue-shifted. Conversely, macromolecular ligands are too large to be confined, resulting in LSPR red-shift [13]. Considering structural modifications and NPs stabilizers, there is still a knowledge gap on the influence of the chemical environments of Au NPs on their electronic properties.

The aim of the following study was to determine the effect of pH on the structure and electronic properties of seeded-growth and citrate-stabilized Au nanoparticles. Therefore, we combined transmission electron microscopy (TEM) and UV-Vis spectroscopy for structural analysis, Fourier-transform infrared (FTIR) spectroscopy, and X-ray photoelectron spectroscopy (XPS) to investigate chemical composition and surface properties with X-ray Absorption Near Edge Structure (XANES) which was used to probe changes in the electronic structure of Au. The XANES spectroscopy is very sensitive to the local electronic structure of the studied element, thus providing information on the local bonding and oxidation state of the specimen. Research reports showed the synergy between size, shape, and molecular ligands of thiol-capped Au NPs using an XAS study [14,15] and investigated it by tracking their electronic and structural dynamics caused by photoexcitation [16]. Others presented a quantitative analysis to determine the number of *d* state vacancies based on the Au L_2_ and L_3_ absorption edge [15,17]. In this work, we have focused on exploring the surface electronic states of pH-dependent sub 10 nm plasmonic nanoparticles and their influence on morphological and chemical structure. Changes in intrinsic structural effects (e.g., morphology) of citrate-capped Au NPs nanoparticles are important in describing their interaction with extrinsic ones (environmental effects). The presented research extends the knowledge about citrate-capped Au NPs structural properties and demonstrates the usefulness of XANES in probing small electronic differences of the nanoparticles induced by the pH reaction.

1. **Experimental**
   1. Chemicals and materials

Tetrachloroauric(III) acid trihydrate (HAuCl_4_⋅3H_2_O), sodium citrate tribasic dehydrate (SC), tannic acid (TA), potassium carbonate (K_2_CO_3_), citric acid monohydrate were purchased from Sigma-Aldrich (St. Louis, Missouri, USA). All chemicals are of analytical grades and were used without further purification. Mili-Q water was used for all procedures.

Commercial thiol-capped Au nanoparticles (Au NPs 4 nm) were purchased from PlasmaChem (GmbH, Berlin, Germany) and used as a reference material.

- 1. Synthesis of Au nanoparticles

Au NPs were synthesized using the reversed Turkevich method [4]. The method involves the reduction of an aqueous solution of HAuCl_4_ using sodium citrate (SC) as a capping and reducing agent. A small amount of tannic acid (TA) was added to increase the reduction kinetics and enhance the formation of Au NPs. Briefly, 6.6 mM of SC and 2.5 mM of TA were dissolved in Mili-Q water. Subsequently, the solutions were mixed together and heated to 70°C. This was followed by the addition of 1 mL of 25 mM HAuCl_4_⋅3H_2_O (Fig.1). The solution's color turned black instantly. After 5 minutes, the reaction was completed based on the ruby-red solution color, indicating that gold ions (Au^3+^) turned into neutral gold atoms (Au^0^). The final product is labeled as Au NPs SC+TA.

Further, to change the chemical surroundings of Au NPs, this synthesis was modified by the addition of 1 mL 0.15 M K_2_CO_3_ and 0.15 M citric acid to SC+TA solution (before the precursor addition), labeled as Au NPs K_2_CO_3_ and Au NPs citric acid, respectively. Another approach was based on heterogenous nucleation (so-called seeding) by two-step introduction (0.5 mL) of the precursor after 10 minutes (labeled as Au NPs 2 inj. seeding). All synthesized Au NPs suspensions were characterized by different colors, from pink to violet (see, Fig.1).


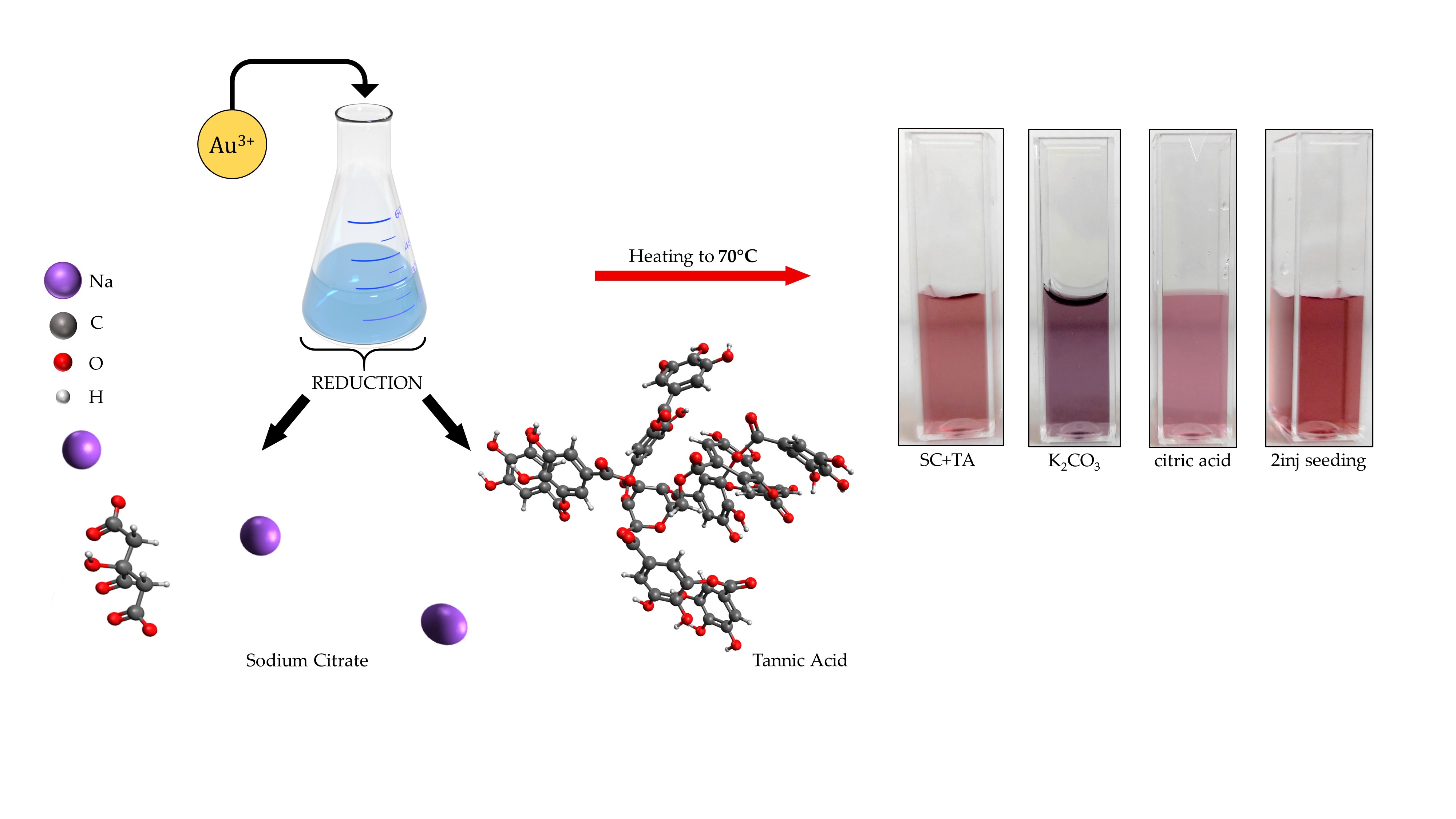
**Fig. 1** Schematic representation of Au NPs synthesis using the Turkevich method.

Finally, to minimize the number of aggregates and obtain an even distribution of nanoparticles, solutions were poured into testing tubes and centrifuged (Pro Analytical C4000, Centurion Scientific Ltd) at 10,000 rpm for 10 minutes. Next, supernatants were transferred to glass dishes and stored in the dark at 4°C. Table 1 summarizes the list of samples synthesized under different reaction parameters.

**Table 1** List of samples subjected to different chemical environments

| **Sample Name** | **Reaction parameters** |
| --- | --- |
| Au NPs SC+TA | No changes during synthesis |
| Au NPs K_2_CO_3_ | Addition of 1 mL 0.15 M K_2_CO_3_ to capping agent (SC+TA) |
| Au NPs citric acid | Addition of 1 mL 0.15 M citric acid to capping agent (SC+TA) |
| Au NPs 2 inj. seeding | Addition of 500 µl of HAuCl_4_ precursor to the capping agent, the process was repeated after 10 minutes |

- 1. Characterization methods

The size and structure of Au NPs were assessed by Transmission Electron Microscope (TEM) in Bright Field (BF) mode. Each Au NPs solution was drop-coated onto a 200 mesh copper grid. The imaging was performed on a Tecnai G2 F20 instrument equipped with an FEG gun and operated at 200 kV accelerating voltage. The images were analyzed to determine the size of both spherical and non-spherical nanoparticles by measuring their area using ImageJ software tools, from which the equivalent diameter was calculated. The data were then converted and further analyzed in Origin 2019b by providing histogram data based on 120 NPs.

The LSPR absorption was determined by UV-Vis spectroscopy. UV-Vis spectra were acquired with an Avantes AvaSpec spectrophotometer in the range of 400 to 900 nm with 1 nm spectral resolution and then evaluated in Origin 2019b.

The surface chemical composition of Au NPs was initially examined by infrared spectroscopy. Attenuated Total Reflectance–Fourier Transform Infrared spectroscopy (ATR–FTIR, Nicolet iS 5, Thermo Fisher Scientific, USA) was performed using the diamond crystal. The measurement was done in a spectrum between 4000 and 400 cm^-1^ and was obtained by averaging 32 scans with a resolution of 4 cm^-1^. Aqueous solution of Au NPs was dropped on the substrate and left to dry until the water evaporated by observing the attenuation of absorption peak from water. Afterwards, spectra were processed in the Origin2019b software.

The synthesized Au NPs were analyzed using X-ray Photoelectron Spectroscopy (XPS) under ultra-high vacuum conditions to determine their surface elemental composition and chemical states. To address the low concentration of the Au in the NPs suspension, all samples were treated with a vacuum evaporator before the measurements to control the evaporation of the solvent. The X-ray photoelectron spectra were measured on a Prevac photoelectron spectrometer equipped with a hemispherical VG SCIENTA R3000 analyzer using a micro-focused and monochromatic AlKα X-ray source (1486.6 eV, spot size: 200 µm). The operating pressure in the analysis chamber was sustained at ~1⋅10^-8^ mbar. The pass energy was set to 100 eV, with a step size of 0.15 eV, to record high-resolution spectra of the C 1s, O 1s, and Au 4f regions for each sample. The binding energy scale for all the samples was referenced to the C 1s core level at 285.0 eV, attributed to hydrocarbon contaminations.

The high-resolution spectra were curve-fitted with a set of component peaks to separate the photoemission signal originating from distinct elemental or chemical states. This curve-fitting was performed using a Simplex optimization algorithm in CasaXPS software (version 2.3.26). Following the subtraction of the Shirley-type background, the core-level spectra were decomposed into their components using a mixed Gaussian/Lorentzian function peak shape. The same fitting parameters and constraints were consistently applied across all samples. Specifically, the full width at half maximum (FWHM) values were constrained to a maximum of 2.2 and, for individual peaks, were restricted to minimal variation to ensure consistency. The Au 4f region was fitted with a doublet resulting from the spin-orbit coupling of the 4f_7/2_ and 4f_5/2_ states, with an energy separation of 3.7 eV and a fixed area ratio of 4:3. The full width at half-maximum height was maintained at 1.3 ± 0.1 eV for both the Au 4f components. Therefore, the surface chemical composition of the samples was determined based on the curve-fitted peak areas, relative sensitive factors and binding energies of Au 4f, O 1s, and C 1s photoelectron peaks.

X-ray absorption spectra (XAS) measurements were performed at the SuperXAS beamline of the Swiss Light Source at Paul Scherrer Institute, Villigen, Switzerland. The X-ray beam delivered by the 2.9-T super bending magnet source was collimated by a Si-coated mirror and subsequently monochromatized. The incidence beam was monitored with the ionization chamber and the X-ray fluorescence from the sample was acquired, with the SDD detector. In order to reduce the scattering radiation that enters the detector, the Ga filter was used. Spectra were measured around the Au L_3_-edge (11.919 keV) in the region of 11.735-12.73 keV. In the region of interest (around the edge), an energy step size of 0.5 eV was used, with a measurement time of 5 seconds per point. 10 µm Au foil spectra were collected simultaneously for internal energy calibration based on the first derivative. Samples were measured in quartz capillary tubes, with a 10 μm wall thickness and an outside diameter of 2 mm. To enable quantitative comparison and analysis of spectra independently of the experimental conditions (e.g. sample concentration), the resulting raw data were pre-processed using Athena software [18]. The normalization procedure consisted of two main steps: (1) fitting the pre-edge baseline region and (2) fitting the post-edge region. In the pre-edge region, a linear polynomial was fitted and subtracted from the data, while in the post-edge region, a quadratic polynomial was applied to achieve an edge jump of 1. The pre-edge and post-edge ranges were consistently maintained across all samples at -180 to 60 eV and 135 to -820 eV to E_0_ (11.919 keV), respectively.

Theoretical calculations of X-ray absorption spectra and density of states (DOS) were conducted using the FEFF9.6 code [19]. FEFF9.6 is a self-consistent multiple-scattering code, which allows the simulation of the electronic structure of materials based on the structural information as input files. All calculations were performed using the FMS (Full Multiple Scattering) approach preceded by Self Consistency Field calculations (SFC) for muffin tin atomic potentials and with the Hedin–Lundqvist as an exchange potential. FEFF calculations were performed for scattering radii of 6Å, using atom distributions and arrangement derived from Au (face-centered cubic – fcc) structure, which were taken from the WebATOMS database.

1. **Results and discussion**
   1. Structural analysis

The reversed Turkevich method is sensitive to various synthesis parameters, such as reaction pH, temperature, heating time, precursor, and capping agent concentrations. Herein, the focus was the reactions’ pH effect, which was found to have an impact on the synthesis of citrate-stabilized Au NPs. Table 2 presents the physicochemical properties, including the solution pH values before and after HAuCl_4_ addition to the synthesis mixture. As shown, the addition of K_2_CO_3_ and citric acid changes the pH, improving the basicity and acidity of the reaction mixture, respectively, which in turn affects the structural and optical properties of the final nanoparticles.

**Table 2** Physicochemical properties of synthesized Au NPs, including pH values before and after the HAuCl_4_ addition as well as LSPR maximum and Full Width at Half Maximum (FWHM) obtained from UV-Vis spectra.

|  | | **Gold nanoparticle type** | | | |
| --- | --- | --- | --- | --- | --- |
|  |  | Au NPs SC+TA | Au NPs citric acid | Au NPs K_2_CO_3_ | Au NPs 2 inj. seeding |
| **pH** | *before HAuCl_4_* | 7.7 | 5.6 | 9.9 | 7.7 |
|  | *after HAuCl_4_* | 6.4 | 5.2 | 9.1 | 6.2 |
| **LSPR (nm)** | | 519 | 529 | 537 | 522 |
| **FWHM (nm)** | | 93.3 | 89.3 | 92.8 | 90.1 |

UV-Vis absorption spectra, presented in Fig. 2a, showed distinct peaks characteristic of the surface plasmon resonance (LSPR) of nano-sized gold. The red shift of the LSPR peak was detected for each variant compared to SC+TA synthesis. The highest shift was found for Au NPs K_2_CO_3_ and the difference between this variant and SC+TA was greater compared to the others. Additionally, Au NPs K_2_CO_3_ displayed the largest nanoparticle size, according to TEM analysis (as described later in the manuscript). Thus, the observed gradual increase in diameters is accompanied by a shift of LSPR maximum towards a higher wavelength.

Besides the pronounced LSPR shift, other significant features observed in the UV-Vis spectra were analyzed. LSPR peak obtained for Au NPs 2 inj. seeding was found to be intense and sharp in contrast to other examined materials. On the other hand, the LSPR band for Au NPs K_2_CO_3_ is damped in comparison to each nanoparticle’s type. Peak broadening was not significant between the samples, according to the determined FWHM values (summarized in Table 2). Gad et al. found that smaller particles show suppression in the LSPR peak due to the reduced mean free path of the electrons, while the larger nanoparticles exhibit amplified scattering peaks, which significantly broaden the LSPR peak, owing to a higher optical cross-section of NPs [20,21].


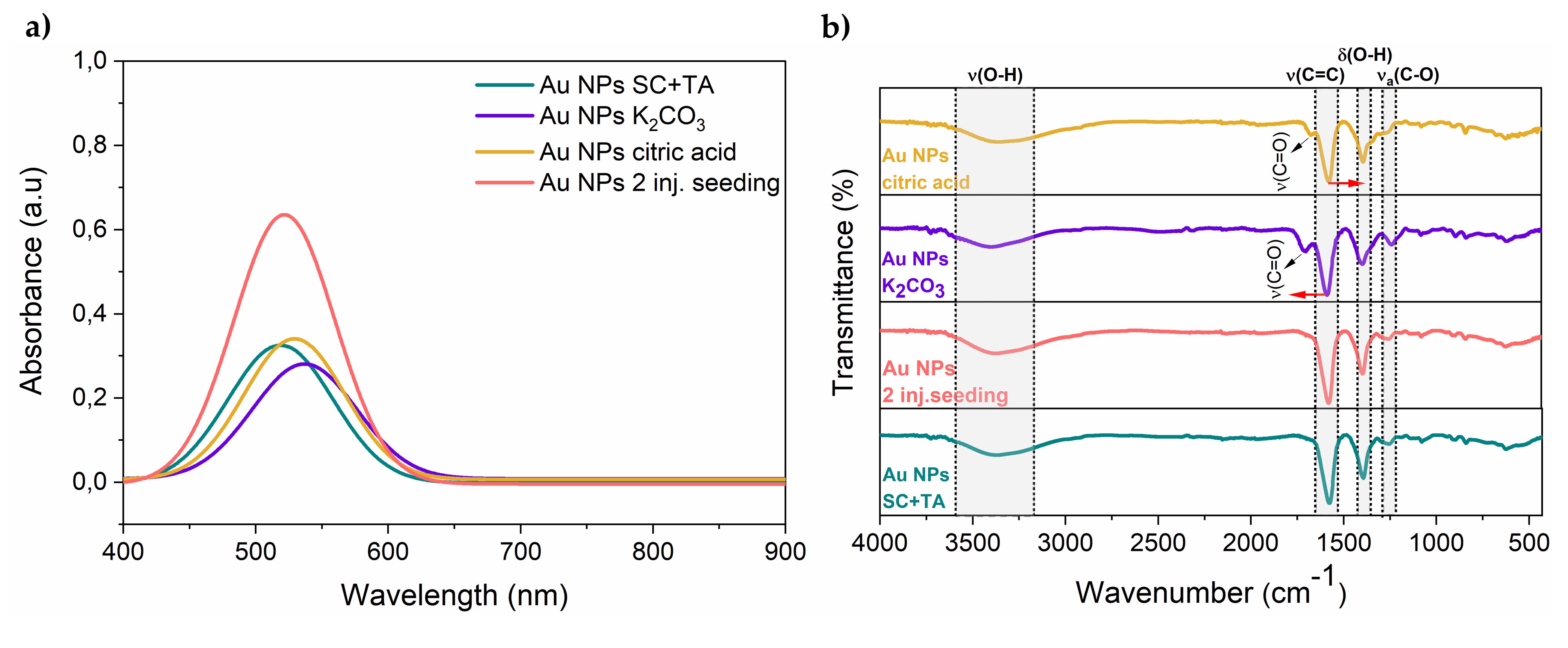
**Fig. 2** Effect of synthesis conditions on properties of Au NPs based on: a) UV-Vis spectra and b) FITR spectra collected for Au NPs synthesized at different chemical environments. The light grey area with dashed lines and the corresponding bold description represent characteristic bands. Red arrows show the peak shift direction. Abbreviations: ν – stretching, ν_a_ – asymmetric stretching, δ – bending.

According to Mie's theory, the position of the absorption peak, its intensity, and FWHM are affected by the nanoparticle’s size, the density of conduction electrons upon surface plasma particles, and the dielectric constant of nanoparticles [20,22]. Herein, both bigger and smaller nanoparticles showed relatively higher peak suppression followed by peak shift. In the case of seeded Au NPs, the highest absorption peak was detected as a result of the sequential addition of precursor to the capping agent.

Due to its high sensitivity to the vibration of chemical bonds in functional groups, FTIR spectroscopy is a method of choice to study influence of pH on surface chemistry of Au NPs. Figure 2b presents FTIR spectra collected for all synthesized samples, showing characteristic peaks for unmodified Au NPs at 3370, 1580, 1395, and 1257 cm^-1^, indicating ν(O–H), ν(C=C), δ(O-H), and ν_a_(C–O) bands, respectively [23,24]. The O–H stretching and bending vibrations of O–H bonds arise from the solvent (water) and citrate molecules. However, peak arisen from 1395 cm^-1^ can be attributed to the presence of tannic acid [25]. The C–C and C–O groups represent distinct peaks for adsorbed citrate molecules for all Au NPs variants. Moreover, it was observed that altering the chemical environment of the capping agent influenced both the intensity and position of characteristic bands with respect to native Au NPs sample. In particular, the intensity of the peak attributed to C=C stretching is lower for Au NPs K­_2_CO_3_ and citric acid samples compared to Au NPs SC+TA. Furthermore, these peaks are shifted to the left (~8 cm^-1^) and right (~ 5 cm^-1^), respectively, compared to native NPs variant. Such variation confirms the protonation and deprotonation of carboxylate groups [26,27]. The spectrum for Au NPs 2 inj. seeding resembles the spectrum recorded for SC+TA. However, it also demonstrates slightly lower intensity in C=C stretching and O–H bending. Additional stretching bands of C=O were found at 1701 cm^-1^ and 1675 cm^-1^ for Au NPs K_2_CO_3_ and Au NPs citric acid, respectively, indicating the presence of carboxyl groups in phenolic structures [28]. These peaks can also correspond to various hydrogen bond configurations and sodium interactions [26,27,29]. Peak attributed to TA showcased significantly lower intensity for Au NPs K_2_CO­_3_ and citric acid opposite to native Au NPs, indicating that O–H groups derived from tannic acid oxidized to C=O [25]. The peak assigned to ν_a_(C–O) was found to be more pronounced for Au NPs K_2_CO_3_ and attenuated for Au NPs citric acid in comparison to native sample. The hydroxide absorption on Au NPs was not observed after the pH increase, which proves the absence of Au–OH vibration at 520 – 580 cm^-1^[26,27]. In alkaline conditions, citrate ions bind to Au NPs surface in their deprotonated form (Cit^3-^). The primary interaction occurs through the central carboxylate group, while one or both terminal carboxylate groups may also absorb to the surface, leading to mono or bidentate citrate binding [29]. Thus, introducing ions derived from K_2_CO_3_ and C_6_H_8_O_7_ can affect the reorganization of the surface coverage groups on Au surface.


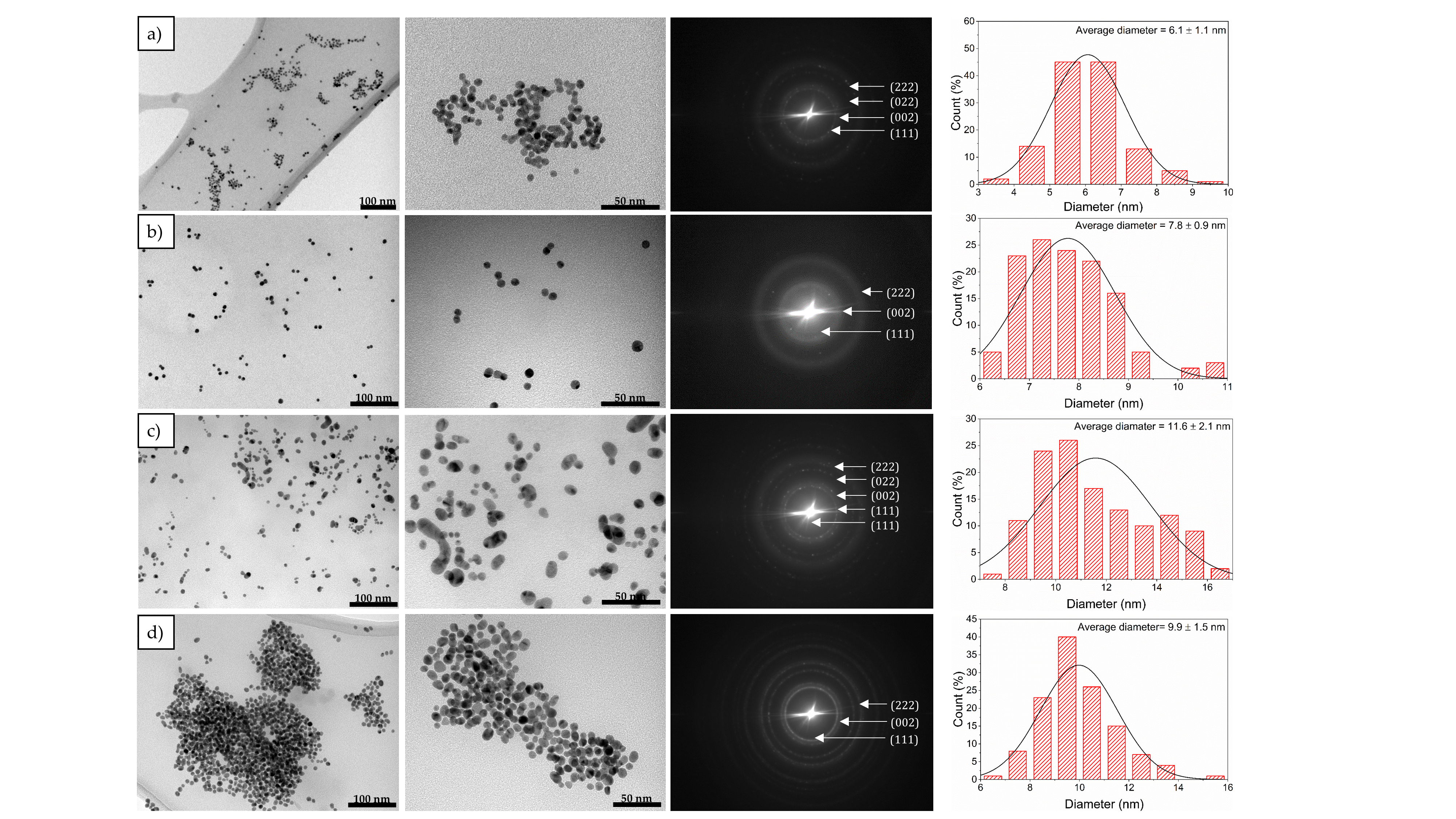
**Fig. 3** Representative TEM micrographs together with size distribution profiles of Au NPs synthesized at different synthesis parameters: a) Au NPs SC+TA, b) Au NPs 2 inj. seeding,  c) Au NPs K_2_CO_3_, and d) Au NPs citric acid. Particle size distribution was fitted with a normal distribution function.

Bright field (BF) TEM images, shown in Fig. 3, reveal the expected quasi-spherical shape of the synthesized nanoparticles. The NPs obtained from SC+TA synthesis were in the size of 6.1 ± 1.1 nm and displayed uniform distribution with some visible agglomeration sites. A larger size (7.8 ± 0.9 nm) was achieved for Au NPs 2 inj. seeding (see Fig.3b), but contrarily to Au NPs SC+TA, presented a monodisperse distribution. After the addition of K_2_CO_3_ to the capping agent, NPs doubled in size (11.6 ± 2.1 nm), showing similar morphology to Au NPs SC+TA, and exhibiting some oblong NPs. The size distribution profile of Au NPs K_2_CO_3_ was considerably broader compared to other samples, suggesting a less efficient interaction between the gold precursor and the capping agent. The addition of citric acid also resulted in an increase in particle size (9.9 ± 1.5 nm) compared to Au NPs SC+TA, but the nanoparticles formed tightly-bounded and spherical agglomerates. It was found that that nonspherical nanoparticles (spheroids) and aggregates often appear in spherical nanoparticle synthesis caused by chemical reduction [30]. Corresponding to all Au NPs surface morphology, selected area electron diffraction (SAED) patterns showed several diffraction rings, which confirmed the nanocrystalline origin of Au NPs (Fig. 3). Those patterns represent the sum of different single-crystallized Au NPs, which exhibit different, but randomly-oriented crystal planes. The patterns were indexed according to (111), (002), (022), and (222) reflections of fcc gold.

Based on microscopic and spectroscopic observations, the NPs size and agglomeration could be associated with the pH of each synthesis variant. The morphology of Au NPs SC+TA, for which the reaction mixture has slightly acidic values (pH~6.4), exhibits the highest uniform size distribution of the produced nanoparticle among all tested variants, which agrees with the literature reports [22,31]. A decrease in pH towards acidity caused strong agglomeration of nanoparticles, while the pH increase resulted in the formation of monodisperse NPs. Figure 4 illustrates the effect of pH on the reactivity of the gold precursor and the reaction mechanism (presumably the nucleation process), affecting the formation of Au NPs. In particular, the basic environment results in Au NPs that are larger in size, which is also translated into larger shifts of the LSPR peak maximum (i.e., from 519 to 537 nm). On the contrary, a slightly acidic environment (pH ~ 6) produces smaller size and narrowly distributed Au NPs. Citrate ions (C₆H₅O₇³⁻) act as a reducing agent, converting Au^3+^ species into Au^0^, and also as a protective agent that stabilizes the formed nanoparticles via electrostatic repulsion [22]. Following Le Chatelier’s principle, an increase in proton concentration within the solution leads to a decrease in citrate’s tendency to oxidize. This, in turn, results in a simultaneous decrease in the availability of electrons for the reduction of AuCl_3_ and, consequently, the formation of Au NPs [22]. Moreover, the coordination bonding between the gold surface and the absorbed citrate molecule involves carboxyl oxygen atoms, which provide the electron pairs necessary for the formation of covalent bonds [32,33]. Here, as a reducer, we used a combination of both SC and TA, in a 3:1 concentration ratio, where TA responds mainly to the redox reaction of gold together with SC. Thus, the addition of K_2_CO_3_ and citric acid to the SC and TA combination led to the increase of protonation and deprotonation of Au NPs solution, respectively, increasing the size and changing the chemical conformation of the absorbed molecules on the surface. In addition, citrate can coordinate with gold through different binding geometries, i.e., bridging or chealating conformation, mainly influenced by carboxyl groups [26,29]. Nonetheless, this intermolecular interaction requires the diffusion of citrate to the gold surface in a fully deprotonated state of carboxyl groups, since the protonation form is not favorable due to electrochemical impediments [32,33]. As described above, the pH value affects the binding affinity between gold NPs and functional groups, which in turn, significantly impacts the size and surface coverage of the studied nanoparticles. This leads to variations in LSPR absorption spectra. Nevertheless, based on the obtained results, it is difficult to clearly establish a model illustrating the correlation between pH value and the LSPR shift.



The presence and conformation of surface-absorbed groups around the nanoparticle may also contributed to this shift, as well as its significant relation with the size of NPs. Toh et al. reported that Au nanorods conjugated to 11-mercaptoundecanoic acid (MUA) modulated the LSPR phenomenon at physiological conditions (pH ~7.49) as a result of electrical charges formed on the surface after protonation/deprotonation of carboxylic groups [34]. In our study, Au NPs synthesized in a strong base environment (pH ~9) exhibited the highest redshift of the LSPR peak, demonstrating the impact of adsorbed molecules’s conformation on the gold surface.

**Fig. 4** Position of LSPR peaks as a function of Au NPs diameter (in yellow) and pH levels (in purple) after precursor addition. LSPR vs Au NPs size was fitted with linear function (yellow).

Importantly, the protonation of citrate ions caused by pH has also an impact on the strong reduction potential of Au^3+^, which in turn affects the surface morphology of Au NPs [35]. Generally, the inverse Turkevich synthesis relies upon the formation of intermediate acetone-dicarboxylate (ADC), which improves the Au NPs dispersity and is also responsible for fast nucleation. Furthermore, pH is strictly related to the electrostatic stabilization of Au NPs and the reactivity of the precursor during nucleation [22,35]. In acidic conditions, citrate protonation leads to decreased stability, whereas in alkaline environments, Au ions exhibit reduced reactivity owing to the formation of hydroxide complexes [4]. Herein, Au NPs citric acid (as seen in Fig. 3d) were strongly aggregated, which can be explained by poor electrostatic stabilization. While K_2_CO_3_ addition could reduce the reactivity of the precursor and stability during Au NPs nucleation. High uniformity and very low size dispersity of nanoparticles were observed for Au NPs SC+TA and 2 inj. seeding variant, obtained at a similar, slightly acidic environment, which in this case may be involved with stable precursor reactivity and electrostatic interactions [22]. Thus, the higher pH of the Au NPs solution may affect the formation of a higher number of citrate ions (C_6_H_5_O_7_^3-^), which is undesirable in Au nucleation. Here, pH ~ 6 can be treated as an optimal value to balance both reactivity and promotion of fast nucleation [36]. Similar to the Turkevich method, inverse Turkevich synthesis can be demanding due to a difficult nucleation mechanism resulting in broad size and shape distribution [37,38]. SC, when mixed with aqueous HAuCl_4_ at high temperature, is oxidized to sodium acetone dicarboxylate, while gold precursor to AuCl. Depending on the pH reaction, AuCl_4_^-^ is hydrolyzed to other forms of auric ions, for instance, at pH 6.2 – AuCl_3_(OH)^-^ and for pH 8.1 – AuCl(OH)_3_^-^, and their reactivity decreases with the pH increase. Due to high number of those ions, the inhomogenous nucleation may occur. In our study, we have not detected the Au–OH vibrations in ATR-FTIR measurements, but the presence of AuCl (OH)_3_^-^ ions and their surface conformation could explain the broad size distribution in Au NPs K_2_CO_3_ [20].

The XPS measurements provided insight into the surface chemical composition of Au NPs prepared in this study. The collected high-resolution C 1s, O 1s, and Au 4f spectra are comparatively shown in Suppl. Mat, Figure S1-S3. The atomic contribution and core-level binding energies of surface species are summarized in Table 3. The main Au 4f signals appear as doublet, with Au 4f_7/2_ and Au 4f_5/2_ peaks well-separated at 83.9 and 87.6 eV, respectively. These values are characteristic binding energies for metallic Au atoms [39]. In the case of the Au NPs citric acid sample, a second doublet is identified at 85.0 eV and 88.6 eV, which can be attributed to the Au^+^. It should be noted that due to the elemental composition of the outermost atomic layers and the vivid presence of capping agent, the Au signal is attenuated in comparison to the literature data [40,41]. Nonetheless, XPS analysis confirms the reduction of the Au (III) to Au (0) and the formation of Au NPs surrounded by the organic shell in each sample. The C 1s core level spectra were fitted with four distinct components: 285.0 eV, 286.2 eV, 287.7 eV and 288.6 eV. The first strong peak is assigned to aliphatic carbons C–C, C–H, and/or α-carbons (CH_2_) [27,42]. The second peak corresponds to carbon atoms present in C−O groups [26,27], whereas the third peak is attributed to the gold-coordinated carboxylates (COO−Au) or C=O and O–C–O moieties [26,27]. The signal with the highest binding energy is indicative of carboxylic functionalities: COOH or COO^−^ [26,27,43]. The first deconvoluted peak contributes to the citrate molecules but could also be attributed to adventitious carbon contamination present in the XPS system [26,27]. The O 1s spectrum exhibited three peaks at 531.5 eV, 532.8 eV, and 535.5 eV, ascribed as O=C, O–C, (H_2_O)_ads_, respectively, which bound in the citrate structure [28]. The atomic content of carbon and oxygen increases for Au NPs citric acid in relation to Au NPs SC+TA, enriching in CH_2_/C–OH and C–O groups. For Au NPs K_2_CO_3_, the content of COOH and C–O decreased compared to native NPs. However, the amount of CH_2_­/C–OH and C=O have arisen. A similar effect was observed in Au NPs 2 inj. seeding. This may suggest the presence of acetoacetate, which is the majorly oxidized species of citrate and has the ability to be desorbed from the NPs surface. It was found that increasing the pH ~9 decreases the number of acetoacetates, making the citrate ions a primary organic compound on the Au NPs surface [26]. Here, the Au NPs K_2_CO_3_ with the pH ~9 showed a growing number of C=O groups derived from COOH hydrogen bonds, compared to Au NPs SC+TA and their presence is also confirmed by IR frequency at 1701 cm^-1^. Despite the findings published by Park et al., these results indicate the presence of acetoacetate on samples in an alkaline environment. Interestingly, the number of those groups showed in XPS and FTIR as well as their intensity was attenuated for Au NPs citric acid (pH ~5) with respect to unmodified NPs (pH ~6). Grys et al. showed that citrate anions under pH<6.8 present bidentate bridging of central carboxylate, while for pH higher than 6.8, unbound carboxylates are found. At lower pH, citrate coverage is reduced, and the ions can span the nanogap [29].

**Table 3** Atomic contributions of surface species in the citrate-capped Au NPs determined by XPS (binding energies in eV given in parentheses). Results are presented as the percentage values of a given element relative to the total element sum.

| Sample | **Surface composition (at.%)** | | | | | | | | | | | |
| --- | --- | --- | --- | --- | --- | --- | --- | --- | --- | --- | --- | --- |
|  | **C 1s** | | | | | **O 1s** | | | | **Au 4f** | | |
|  | C-C, C-H  (285.0) | C-O  (286.2±0.2) | C=O,  O-C-O  COO-Au  (287.7±0.2) | COOH,  COO^-^  (288.6±0.2) | C*_total_*  (at. %) | O=C  (531.5±0.1) | O-C  (532.8±0.1) | H_2_O_ads_  (535.5±0.2) | O*_total_*  (at. %) | Au^0^  (83.9±0.1) | Au^+^  (85.0) | Au*_total_*  (at. %) |
|  | C/C*_total_* (%) | | | |  | O/O*_total_* (%) | | |  | Au/Au*_total_* (%) | |  |
| **Au NPs**  **SC+TA** | 51.3 | 17.0 | 3.9 | 27.9 | 50.78 | 56.3 | 29.6 | 14.1 | 49.13 | 100 | - | 0.09 |
| **Au NPs**  **K_2_CO_3_** | 50.8 | 21.1 | 3.6 | 24.5 | 52.86 | 62.2 | 24.8 | 13.1 | 47.04 | 92.6 | 7.4 | 0.10 |
| **Au NPs**  **citric acid** | 48.9 | 18.6 | 4.2 | 28.3 | 51.54 | 40.3 | 48.6 | 11.0 | 48.44 | 100 | - | 0.02 |
| **Au NPs**  **2 inj. seeding** | 47.0 | 17.5 | 8.9 | 26.6 | 49.16 | 62.5 | 21.7 | 15.8 | 50.76 | 100 | - | 0.08 |

- 1. X-ray absorption spectroscopy analysis

X-ray absorption spectroscopy at the Au L_3_-edge was used to probe changes in the electronic structure and local environment of gold sites in studied nanoparticles. The X-ray absorption near edge structure (XANES) spectra of all Au NPs variants and, for comparison, reference samples: Au foil and commercial Au NPs (4 nm in diameter), are plotted in Fig. 5a. Collected XANES spectra are typical for the fcc Au structure, showing three characteristic features within ~40 eV above the absorption edge (11919 eV), marked with the vertical dashed lines. The shape and resonance peak positions were similar to bulk material (Au foil), which indicates minor changes in the local environment of the Au atom [44,45]. To support the analysis of those changes, each Au NPs spectrum was subtracted from bulk one to obtain the difference signal plot (Fig.5b). The spectral feature observed at ~11920 eV, the so-called white line, is associated with the transition of *2p_3/2_* electrons to the lowest unoccupied electronic states (*d*-states) [46]. Generally, *5d* orbitals in metallic Au atoms are fully occupied. However, the observed small white line in bulk material (Au foil) results from *s-p-d* hybridization [47]. In this case, a more intense white line reflects the increase in the *d*-hole population related to the hybridization. It has been shown that the intensity of the resonance at the threshold can be used to study the *d*-charge redistributions in various gold samples [10,11,47], being the interplay between the nano-size and surface functionality (tuned by capping agent) effects. The size of Au nanoparticles corresponds to a certain number of surface and bulk atoms. Namely, the smaller the size, the greater the fraction of atoms located on the surface. For instance, the Au NPs SC+TA have a size corresponding to approximately 7000 atoms, which, according to calculations presented in [48], equals ~12.5% of surface atoms. Therefore, experimental XANES spectra are a combination of spectra originating from both bulk and surface, containing averaged information about unoccupied states in Au *d*-bands.

It was found that Au NPs SC+TA showed a minor decrease in the first resonance peak and a negative difference, indicating the *5d* electron gain (or loss of *5d* holes). Similarly, commercial Au nanoparticles (thiol-capped) displayed a small decrease in the white line. However, this was accompanied by a small but noticeable shift in the absorption edge towards higher energy compared to the bulk gold. These observations are consistent with the previous reports on thiol-capped Au NPs [45,49]. The opposite effect was observed in XANES spectra collected for Au NPs K_2_CO_3_ and citric acid, namely an increase in the white line, suggesting *5d* electron loss. Neither an increase nor a decrease in the edge peak was observed for Au NPs 2 inj. seeding, which showed a similar resonance pattern to the bulk material. Nevertheless, a small decrease was detected in the third feature (~11945 eV), meaning the gain of *5d* electrons. It should be noted that the features above the white line (labelled as II and III in Figure 5a), correspond to the resonances characteristic of the extended local structure of the fcc Au metal. Their shift towards higher energies and broadening is associated with lower coordination numbers, higher structural disorder, and smaller bond distances in nanoparticles [45,49]. Therefore, a small deep between ~11942 to 11949 eV observed in difference spectra in Figure 5b may be correlated with the increased size of studied Au nanoparticles. It has been reported that non-capped Au NPs reduce their surface energy by rehybridizing the surface and core Au atoms, thereby enhancing the *d-d* interaction within the core. In contrast, nanoparticles capped with thiol ligands minimize surface energy by promoting strong Au-S bonding [11]. Thus, in commercial thiol-capped nanoparticles, this interaction might be attributed to the high Au-S affinity. In the case of SC+TA and 2 inj. seeding variants, the *5d* gain may be explained by a slightly acidic environment around the Au NPs caused by the protonation of carboxyl groups. Contrarily, the number of unoccupied *d* orbitals for Au NPs K_2_CO_3_ and citric acid can be addressed to the deprotonation of those groups, which might have started before the HAuCl_4_ injection. The studied Au NPs have sizes ranging from 6 to 11 nm, as determined by TEM, indicating that size-related effects should have a similar impact in the XANES region. According to Zhang et al., nanosize effects are predominant in Au NPs smaller than 4 nm [11]. Therefore, in the studied samples, the observed changes in the electronic structure are likely due to interactions with the capping molecules. However, size-related effects cannot be entirely ruled out. In nanoscale materials, the interplay between the size effects (quantum confinement of electrons) and surface effects (increasing contribution of surface atoms) impacts both the structure and electronic properties of nanoparticles.





**Fig. 5** Effect of synthesis conditions on properties of Au NPs based on XAS spectral analysis: a) Normalized Au L_3_-edge XANES spectra of all Au NPs variants, compared with bulk Au and commercial Au NPs, b) Difference signal obtained by subtracting XANES spectrum of each Au NPs variant from Au foil (vertical spectra offset), c) Comparison of the experimental spectrum with the FEFF simulated spectrum for bulk Au with calculated projected density of electronic states reflecting Au orbital composition.

Analysis of experimental spectral features was supported with DOS (density of states) theoretical calculations based on *ab initio* FEFF 9.6 code. Calculations were performed for scattering radii of 6Å, using atom distributions and arrangement derived from Au fcc structure. To align the theoretical calculations with the experimental data, the simulated XAS profile had to be shifted in the energy value to fit the experimental data. The adjustment is necessary in order to account for the absolute energy mismatch given by the calculations. The comparison of theoretical and experimental spectra is shown in Fig 5c. It should be noted that in the near-edge region, the simulated spectrum reproduces all significant features of the experimental spectrum. The observed discrepancies in intensities of the spectral features between the experiment and the FEFF simulations are due to the limitations of the muffin-tin (MT) potential approximation implemented within the FEFF code. Subsequently, the experimental spectrum was scaled to the Fermi energy by subtracting a value determined from the position of the XAS inflection point [50–53]. The calculated *l*-projected DOS functions, namely *s*, *p,* and *d*-states of gold, are plotted in Figure 5c. As shown, the occupied electronic states of metallic gold below the Fermi level consist mainly of *d* orbitals, with small contributions of *s* and *p*-states. On the other hand, a small contribution of *d* orbitals is observed above the Fermi level, which is consistent with the *d^10^* electronic configuration of gold. Unoccupied DOS plots indicate that the weak absorption in the white line region arises from a transition to a hybrid partially unoccupied Au *s*-*d* band. It should be mentioned that although the Au *p*-DOS contribution to the XAS signal is expected to be small due to the dipole selection rules, they may also be involved in hybridization with other orbitals. Therefore, *d*-charge redistribution in Au NPs may be interpreted using the *s-p-d* hybridization model, influenced by the overlap between *d* and *s*/*p* bands.

XANES investigation showed that different chemical environments have a significant impact on the size of nanoparticles and the number of neighbors surrounding Au atoms in Au NPs [54]. Hence, the unoccupied *d* states might been compensated with attached ligands, i.e., carboxyl or hydroxide groups. As explained by Zhang et al., the heightened intensity of the white line observed for Au NPs capped with alkene-thiolate was attributed to an increase in size, compared to the bulk [11]. Furthermore, their findings were supported by theoretical calculations, which confirmed that the percentage of *d* holes increased with the size of the nanoparticles [11]. Our study also demonstrates that the size of Au NPs increases with the number of *d* hole states, aligning with Zhang’s research. Here, the pH level during the synthesis of Au NPs played an additional role in this effect. It is plausible to assume that variations in the conformation of absorbed molecules due to pH levels could also have contributed to the presence of *d* hole states. On the other hand, Behafarid et al. found that changes in the intensity of white line are associated with the interaction between Au NPs and their surroundings (i.e., supports, ligands). However, based on Au NPs functionalized with P2VP ligand, they noted that the decrease in white line peak with decreasing NPs size is unrelated to charge transfer between ligand and NPs [14], attributing these changes mainly to the finite particle size. In this study, we have only focused on changing the chemical environment of the capping agent (organic shell), without introducing or exchanging specific ligands. Additionally, considering the unoccupied *d* states, it was observed that hydrogen chemisorption can generate changes in the XANES spectra, approx. 15 eV above the Au L_3_ edge [15]. In the case of Au NPs SC+TA and 2 inj. seeding, the increased peak intensity may result from transitions in the antibonding orbital of metal-hydrogen bonds or multiple scattering of adsorbed negative hydrogen atoms. Therefore, the intensity of the white line peak induced by pH in Au NPs K­_2_CO_3_ and citric acid may be attributed to the hybridization of molecules absorbed on the surface.

1. **Conclusions**

The presented work showed advanced physicochemical investigation on citrate-capped Au NPs using comprehensive analytical methods to determine the pH dependency of Au NPs. Surface and morphological analysis headed by TEM, FTIR, XPS, and UV-Vis spectroscopy underscored the pH sensitivity of the capping agent, which governs not only the LSPR effect but also impacts the size and surface functionalization of the nanoparticles. Moreover, observed external features of Au NPs were linked to their intrinsic characteristics, where we probed the XANES structure to identify changes in the surface electronic states of the nanoparticles. We have shown that small shifts and broadening of peaks above the edge indicate an increase in size due to structural disorder and smaller bond distances in Au NPs. The results were confirmed by the FEFF model. We have also indicated that the presence of unoccupied *d* states in Au NPs K_2_CO_3_ and citric acid can be attributed to the reorganization of adsorbed molecules on the nanoparticle surface, which was also supported by XPS and FTIR data. Therefore, using XANES complemented by XPS and FTIR, we were able to study surface states of citrate-capped Au NPs in a different chemical environment. The next phase of this research should focus more on developing a molecular dynamics model of the citrate-capped Au NPs to explore the surface chemistry of Au NPs in extreme pH conditions along with their moieties and use EXAFS to refer to already obtained data.

**Acknowledgments**

The work was fully funded by the National Science Centre in Poland under grant number 2020/37/B/ST3/00555. The authors would like to acknowledge Paul Scherrer Institute for the access to the SuperXAS beamline at Swiss Light Source, Switzerland. The work is partially supported under the Polish Ministry and Higher Education project: “Support for research and development with the use of research infrastructure of the National Synchrotron Radiation Centre SOLARIS” under contract nr 1/SOL/2021/2.

**Data availability**

The datasets generated and/or analyzed during the current study are available from the corresponding author upon reasonable request.

**Statements and Declarations**

**Confict of interest**

The authors declare no competing interests.

**References**

1. Baig N., Kammakakam I., Falath W., Nanomaterials: A Review Of Synthesis Methods, Properties, Recent Progress, And Challenges. *Mater. Adv.* **2**, 1821-1871; [https://doi.org/10.1039/D0MA00807A](https://doi.org/10.1039/D0MA00807A%20) (2021).
2. Pretzer L.A., Nguyen Q.X., Wong M.S., Controlled Growth Of Sub-10 Nm Gold Nanoparticles Using Carbon Monoxide Reductant. *J. Phys. Chem. C*. **114**, 21226–21233; <https://doi.org/10.1021/jp107945d> (2010).
3. Jiang Y., *et al*., The Interplay Of Size And Surface Functionality On The Cellular Uptake Of Sub-10 Nm Gold Nanoparticles. *ACS Nano.* **9**, 9986–9993; <https://doi.org/10.1021/acsnano.5b03521> (2015).
4. Piella J., Bastús N.G., Puntes V., Size-Controlled Synthesis Of Sub-10-Nanometer Citrate-Stabilized Gold Nanoparticles And Related Optical Properties. *Chem. Mater.* **28**, 1066–1075; <https://doi.org/10.1021/acs.chemmater.5b04406> (2016).
5. Thanh N.T.K., Maclean N., Mahiddine S., Mechanisms Of Nucleation And Growth Of Nanoparticles In Solution. *Chem. Rev.* **114,** 7610–7630; <https://doi.org/10.1021/cr400544s> (2014).
6. Garcia M.A., Surface Plasmons In Metallic Nanoparticles: Fundamentals And Applications. *J. Phys. D: Appl. Phys*. **44**, 283001; <https://doi.org/10.1088/0022-3727/44/28/283001> (2011).
7. Rossi T.P., Erhart P., Kuisma M., Hot-Carrier Generation In Plasmonic Nanoparticles: The Importance Of Atomic Structure. *ACS Nano* **14**, 9963–9971; <https://doi.org/10.1021/acsnano.0c03004> (2020).
8. Jeon H.B., Tsalu P.V., Ha J.W., Shape Effect On The Refractive Index Sensitivity At Localized Surface Plasmon Resonance Inflection Points Of Single Gold Nanocubes With Vertices. *Sci. Rep.* **9**,13635; <https://doi.org/10.1038/s41598-019-50032-3> (2019).
9. Ramallo-López J.M., *et al*., Molecular Conformation Changes In Alkylthiol Ligands As A Function Of Size In Gold Nanoparticles: X-Ray Absorption Studies. *Phys. Rev. B* **74**, 073410; <https://doi.org/10.1103/PhysRevB.74.073410> (2006).
10. Zhang P., Sham T.K., Tuning The Electronic Behavior Of Au Nanoparticles With Capping Molecules. *Appl. Phys. Lett*. **81**, 736–738; <https://doi.org/10.1063/1.1494120> (2002).
11. Zhang P., Sham T.K., X-Ray Studies Of The Structure And Electronic Behavior Of Alkanethiolate-Capped Gold Nanoparticles: The Interplay Of Size And Surface Effects. *Phys. Rev. Lett*. **90**, 245502; <https://doi.org/10.1103/PhysRevLett.90.245502> (2003).
12. He M.Q., *et al.,* Precisely Tuning LSPR Property Via “Peptide-Encoded” Morphological Evolution Of Gold Nanorods For Quantitative Visualization Of Enzyme Activity*. Anal. Chem.* **92**, 7054–7061; <https://doi.org/10.1021/acs.analchem.9b04573> (2020).
13. Oh J.W., Lim D.K., Kim G.H., Suh Y.D., Nam J.M., Thiolated DNA-Based Chemistry And Control In The Structure And Optical Properties Of Plasmonic Nanoparticles With Ultrasmall Interior Nanogap. *J. Am. Chem. Soc.* **136**, 14052–14059; <https://doi.org/10.1021/ja504270d> (2014).
14. Behafarid F., *et al.,* Structural And Electronic Properties Of Micellar Au Nanoparticles: Size And Ligand Effects, *ACS Nano*. **8**, 6671–6681; <https://doi.org/10.1021/nn406568b> (2014).
15. Ohyama J., *et al.,* In Situ Au L3 And L2 Edge XANES Spectral Analysis During Growth Of Thiol Protected Gold Nanoparticles For The Study On Particle Size Dependent Electronic Properties. *Chem. Phys. Lett.* **507,**105–110; [https://doi.org/10.1016/j.cplett.2011.03.020](%20https:/doi.org/10.1016/j.cplett.2011.03.020) (2011).
16. Zamponi F., *et al.,* Probing The Dynamics Of Plasmon-Excited Hexanethiol-Capped Gold Nanoparticles By Picosecond X-Ray Absorption Spectroscopy. *Phys. Chem. Chem. Phys*. **16**, 23157–23163; <https://doi.org/10.1039/c4cp03301a> (2014).
17. Mansour A.N.,Cook Jr J.W., Sayers D.E, Quantitative Technique For The Determination Of The Number Of Unoccupied D-Electron States In A Platinum Catalyst Using The L2,3 X-Ray Absorption Edge Spectra. *J. Phys. Chem.* **88**, 2330-2334; <https://doi.org/10.1021/j150655a029> (1984).
18. Ravel B., Neville M., ATHENA, ARTEMIS, HEPHAESTUS: Data Analysis For X-Ray Absorption Spectroscopy Using IFEFFIT. *J. Synchrotron Radiat*. **12**, 537-541; <https://doi.org/10.1107/S0909049505012719> (2005).
19. Rehr J.J., Kas J.J., Prange M.P., Sorini A.P., Takimoto Y., Vila F., Ab Initio Theory And Calculations Of X-Ray Spectra. *C. R. Phys*. **10**, 548–559; <https://doi.org/10.1016/j.crhy.2008.08.004> (2009).
20. Njoki P.N *et al.,* Size Correlation Of Optical And Spectroscopic Properties For Gold Nanoparticles. *J. Phys. Chem. C.* **111**, 14664–14669; <https://doi.org/10.1021/jp074902z> (2007).
21. Gad G.M.A., Hegazy M.A., Optoelectronic Properties Of Gold Nanoparticles Synthesized By Using Wet Chemical Method. *Mater. Res. Express*. **6**, 085024; <https://doi.org/10.1088/2053-1591/ab1bb8> (2019).
22. Zhu J. *et al.,* Influence Of The pH Value Of A Colloidal Gold Solution On The Absorption Spectra Of An LSPR-Assisted Sensor. AIP Adv. 4:031338; <https://doi.org/10.1063/1.4869615> (2014).
23. Wulandari P., Li X., Tamada K., Hara M., Conformational Study Of Citrates Adsorbed On Gold Nanoparticles Using Fourier Transform Infrared Spectroscopy. *J. Nonlinear Opt. Phys. Mater.* **17**, 185–192; <https://doi.org/10.1142/S0218863508004032> (2008).
24. Tirkey A., Babu P.J., Synthesis And Characterization Of Citrate-Capped Gold Nanoparticles And Their Application In Selective Detection Of Creatinine (A Kidney Biomarker). *Sensors Int.* **5**, 100252; <https://doi.org/10.1016/j.sintl.2023.100252> (2024).
25. Ranoszek-Soliwoda K., *et al.,* The Role Of Tannic Acid And Sodium Citrate In The Synthesis Of Silver Nanoparticles. *J. Nanoparticle Res.* **19**, 273; <https://doi.org/10.1007/s11051-017-3973-9> (2017).
26. Park J.W., Shumaker-Parry J.S., Structural Study Of Citrate Layers On Gold Nanoparticles: Role Of Intermolecular Interactions In Stabilizing Nanoparticles. *J. Am. Chem. Soc.* **136**, 1907–1921; <https://doi.org/10.1021/ja4097384> (2014).
27. Park J.W., Shumaker-Parry J.S., Strong Resistance Of Citrate Anions On Metal Nanoparticles To Desorption Under Thiol Functionalization. *ACS Nano.* **9**, 1665–1682; <https://doi.org/10.1021/nn506379m> (2015).
28. Gubitosa J., *et al*., One Pot Environmental Friendly Synthesis Of Gold Nanoparticles Using Punica Granatum Juice: A Novel Antioxidant Agent For Future Dermatological And Cosmetic Applications. *J. Colloid Interface Sci.* **521**, 50–61. <https://doi.org/10.1016/j.jcis.2018.02.069> (2018).
29. Grys D.B., *et al*., Citrate Coordination And Bridging Of Gold Nanoparticles: The Role Of Gold Adatoms In Aunp Aging. *ACS Nano.* **14**, 8689–8696; <https://doi.org/10.1021/acsnano.0c03050> (2020).
30. Amendola V., Meneghetti M., Size Evaluation Of Gold Nanoparticles By UV-Vis Spectroscopy. *J. Phys. Chem. C.* **113**, 4277–4285; [https://doi.org/10.1021/jp8082425](https://doi.org/10.1021/jp8082425%20) (2009).
31. Yazdani S., *et al.,* Model For Gold Nanoparticle Synthesis: Effect Of pH And Reaction Time. *ACS Omega*. **6**, 16847–16853; <https://doi.org/10.1021/acsomega.1c01418> (2021).
32. Contreras-Trigo B., *et al.,* Slight pH Fluctuations In The Gold Nanoparticle Synthesis Process Influence The Performance Of The Citrate Reduction Method. *Sensors* **18**, 2246; <https://doi.org/10.3390/s18072246> (2018).
33. Monti S., Barcaro G., Sementa L., Carravetta V., Ågren H., Characterization Of The Adsorption Dynamics Of Trisodium Citrate On Gold In Water Solution. *RSC Adv.* **7**, 49655–49663; <https://doi.org/10.1039/C7RA10759E> (2017).
34. Toh Y.R., Yu P., Wen X., Tang J., Hsieh T.S., Induced pH-Dependent Shift By Local Surface Plasmon Resonance In Functionalized Gold Nanorods. *Nanoscale Res. Lett.* **8**, 103; <https://doi.org/10.1186/1556-276X-8-103> (2013).
35. Schulz F. *et al.,* Little Adjustments Significantly Improve The Turkevich Synthesis Of Gold Nanoparticles. *Langmuir* **30**, 10779–10784; <https://doi.org/10.1021/la503209b> (2014).
36. Li C., Li D., Wan G., Xu J., Hou W., Facile Synthesis Of Concentrated Gold Nanoparticles With Low Size-Distribution In Water: Temperature And pH Controls. *Nanoscale Res. Lett.* **6**, 1–10; <https://doi.org/10.1186/1556-276X-6-440> (2011).
37. Xia H., Bai S., Hartmann J., Wang D., Synthesis Of Monodisperse Quasi-Spherical Gold Nanoparticles In Water Via Silver(I)-Assisted Citrate Reduction. *Langmuir* **26**, 3585–3589; [https://doi.org/10.1021/la902987w](https://doi.org/10.1021/la902987w%20) (2010).
38. Goia D. V., Matijević E., Tailoring The Particle Size Of Monodispersed Colloidal Gold. *Colloids Surfaces A Physicochem. Eng. Asp.* **146**, 139–152; <https://doi.org/10.1016/S0927-7757(98)00790-0> (1999).
39. Li X., Li Y., Tan Y., Yang C., Li Y., Self-Assembly Of Gold Nanoparticles Prepared With 3,4- Ethylenedioxythiophene As Reductant. *J. Phys. Chem. B.* **108**, 5192–5199; <https://doi.org/10.1021/jp0356618> (2004).
40. Ouyang R., *et al.,* Bistratal Au@Bi2S3 Nanobones For Excellent NIR-Triggered/Multimodal Imaging-Guided Synergistic Therapy For Liver Cancer. *Bioact. Mater.* **6**, 386–403; [https://doi.org/10.1016/j.bioactmat.2020.08.023](https://doi.org/10.1016/j.bioactmat.2020.08.023%20) (2021).
41. Reinecke B.N., *et al.*, Elucidating The Electronic Structure Of Supported Gold Nanoparticles And Its Relevance To Catalysis By Means Of Hard X-Ray Photoelectron Spectroscopy. *Surf. Sci.* **650**, 24–33; https://doi.org/10.1016/j.susc.2015.12.025 (2016).
42. Joseph Y., *et al.*, Self-Assembled Gold Nanoparticle / Alkanedithiol Films : Preparation, Electron Microscopy, XPS-Analysis, Charge Transport, And Vapor-Sensing Properties. *J. Phys. Chem. B.* **107**, 7406–7413; <https://doi.org/10.1021/jp030439o> (2003).
43. Naumkin G.S.W., Alexander V., Kraut-Vass A., Powell C. J., NIST X-Ray Photoelectron Spectroscopy Database, *NIST Stand. Ref. Database Number* **20**; [https://doi.org/https://dx.doi.org/10.18434/T4T88K](https://doi.org/https:/dx.doi.org/10.18434/T4T88K) (updated 2023).
44. Liu J., *et al.*, Ligand-Stabilized And Atomically Precise Gold Nanocluster Catalysis: A Case Study For Correlating Fundamental Electronic Properties With Catalysis. *Chem. - A Eur. J.* **19**, 10201–10208; <https://doi.org/10.1002/chem.201300600> (2013).
45. Balerna A., *et al.,* Extended X-Ray-Absorption Fine-Structure And Near-Edge-Structure Studies On Evaporated Small Clusters Of Au. *Phys. Rev. B.* **31**, 5058-5065; <https://doi.org/10.1103/physrevb.31.5058> (1985).
46. Guerrero E., *et al.,* Evolution Of The Microstructure, Chemical Composition And Magnetic Behaviour During The Synthesis Of Alkanethiol-Capped Gold Nanoparticles. *Acta Mater.* **55**, 1723–1730; <https://doi.org/10.1016/j.actamat.2006.10.033> (2007).
47. López-Cartes C., *et al.,* Gold Nanoparticles With Different Capping Systems:  An Electronic And Structural XAS Analysis. *J. Phys. Chem. B* **109**, 8761–8766; <https://doi.org/10.1021/jp050184> (2005).
48. Nanda S, Nanda KK. Identifying the Accuracy of Various Approaches for Determining the Fraction of Surface Atoms in a Nanoparticle to Deepen Students’ Understanding of Size-Dependent Properties. *J. Chem. Educ.* **98**, 1982–1987; <https://doi.org/10.1021/acs.jchemed.0c01247> (2021).
49. Sharma S.K., Johannessen B., Golovko V.B., Marshall A.T., X-Ray Absorption Spectroscopy Of Phosphine-Capped Au Clusters. *Inorganics* **11**, 191; <https://doi.org/10.3390/inorganics11050191> (2023).
50. Preston A.R.H., *et al.*, Band Structure Of Zno From Resonant X-Ray Emission Spectroscopy, *Phys. Rev. B.* **78**, 3–6; [https://doi.org/10.1103/PhysRevB.78.155114](https://doi.org/10.1103/PhysRevB.78.155114%20) (2008).
51. Chiou J.W., *et al.*, Charge Transfer In Nanocrystalline- AuZnO Nanorods Investigated By X-Ray Spectroscopy And Scanning Photoelectron Microscopy, *Appl. Phys. Lett.* **90**, 3–6; <https://doi.org/10.1063/1.2738369> (2007).
52. Szlachetko J., Sá J., Rational Design Of Oxynitride Materials: From Theory To Experiment, *CrystEngComm.* **15**, 2583–2587; <https://doi.org/10.1039/c3ce26909d> (2013).
53. Wach A., Sá J., Szlachetko J., Comparative Study Of The Around-Fermi Electronic Structure Of 5d Metals And Metal-Oxides By Means Of High-Resolution X-Ray Emission And Absorption Spectroscopies. *J. Synchrotron Radiat.* **27**, 689–694; [https://doi.org/10.1107/S1600577520003690](https://doi.org/10.1107/S1600577520003690%20) (2020).
54. Akolekar D.B., Foran G., Bhargava S.K., X-Ray Absorption Spectroscopic Studies On Gold Nanoparticles In Mesoporous And Microporous Materials. *J. Synchrotron. Radiat*. **11**, 284-290; <https://doi.org/10.1107/S0909049504001694> (2004).
