# Supplementary figures and images for: Probing electronic-structure pH-dependency of Au nanoparticles through X-ray Absorption Spectroscopy
Source: Sci Rep. 2024 Dec 3;14:30059. doi: 10.1038/s41598-024-81580-y (PMC11615364; doi:10.1038/s41598-024-81580-y)

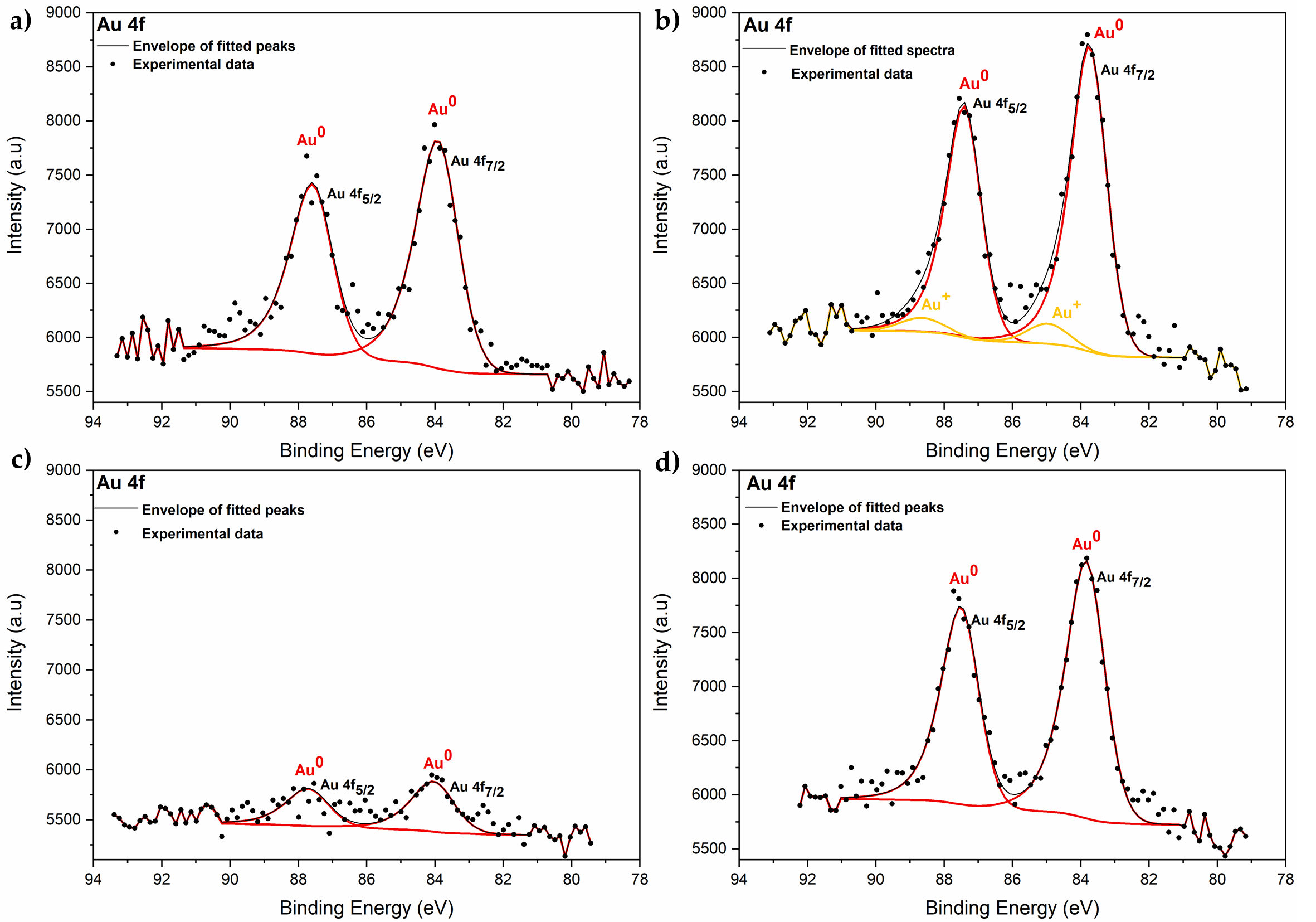

Supplement: Supplementary file 4 — Supplementary Material 4 [file 41598_2024_81580_MOESM4_ESM.tif]

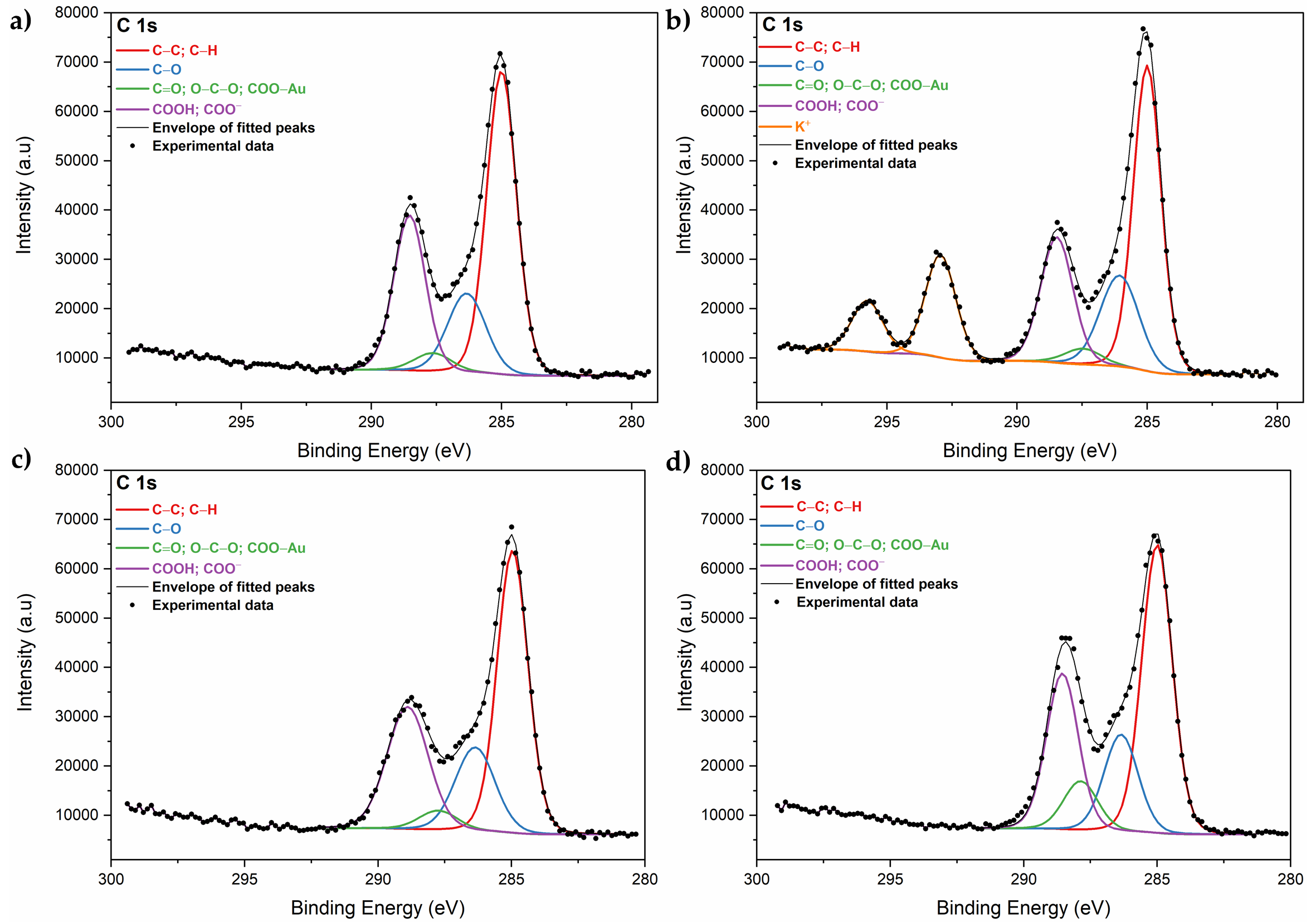

Supplement: Supplementary file 5 — Supplementary Material 5 [file 41598_2024_81580_MOESM5_ESM.tif]

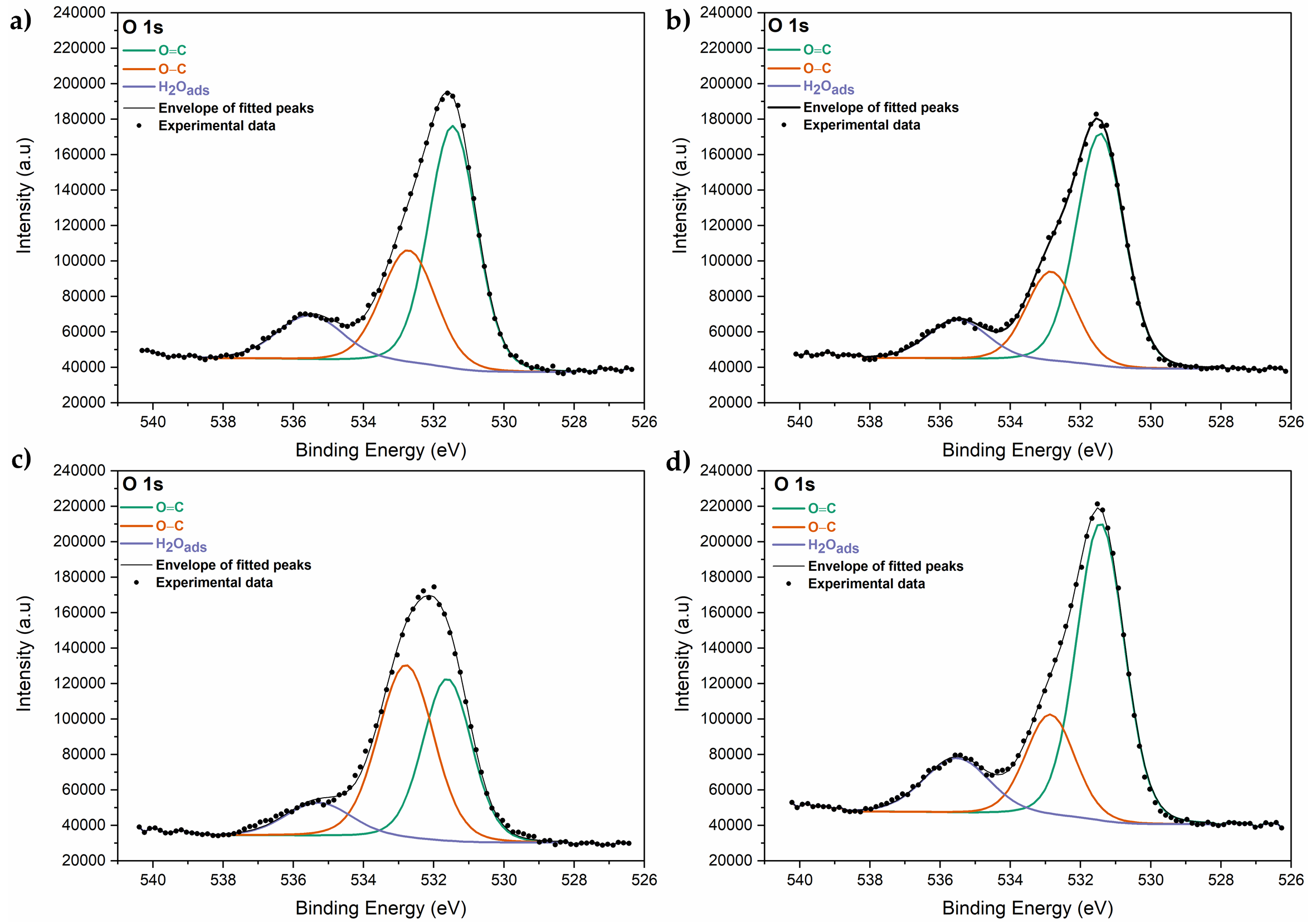

Supplement: Supplementary file 6 — Supplementary Material 6 [file 41598_2024_81580_MOESM6_ESM.tif]
